# Optimizing Mealtime (OPTIMAL): A Pilot Trial of a Person‐Centered Care Program in Nursing Home Care Staff and Residents with ADRD

**DOI:** 10.1002/alz.088393

**Published:** 2025-01-09

**Authors:** Wen Liu, Heather Suh, Kristine N Williams, Elizabeth Galik, Barbara Resnick, Kathleen Buckwalter, Kimberly S VanHaitsma, Barbara Rakel

**Affiliations:** ^1^ The University Of Iowa, Iowa City, IA USA; ^2^ University of Kansas Medical Center, Kansas City, KS USA; ^3^ University of Maryland Baltimore, Baltimore, MD USA; ^4^ University of Iowa, Iowa City, IA USA; ^5^ The Pennsylvania State University, University Park, PA USA

## Abstract

**Background:**

Nursing home (NH) residents with dementia commonly experience mealtime behaviors that negatively impact nutrition and function. Residents do not receive person‐centered mealtime care (PCMC) due to multilevel factors one prioritized modifiable factor is lack of effective PCMC programs. This study aimed to develop a PCMC program and test its feasibility, acceptability, usefulness and preliminary efficacy.

**Method:**

We developed Optimizing Mealtime (OPTIMAL), a multifaceted, evidence‐based, theoretically grounded PCMC program with five components: community stakeholder engagement, environment/policy assessment/modifications, staff education, individualized care planning, and ongoing motivation. A mixed methods design (single‐group repeated measures design followed by stakeholders’ interviews) was used. Repeated measures (resident mealtime resistive behaviors, mealtime functional impairments, eating independence, and food intake amount) were collected at baseline (T1), and at 6‐week (T2) and 12‐week (T3) post baseline.

**Result:**

OPTIMAL was delivered in one NH, where 95 direct care staff received education (age = 45±12.1 years, 13% male, 15% non‐white, 19% Hispanic, 89% experienced challenges with mealtime care), and 17 residents with moderate‐to‐severe dementia were observed (age = 74±7.9 years, 88% male, 12% non‐white, 6% Hispanic). We achieved high recruitment (74%, 77%) and retention (94%, 99%) rates for residents and staff. Stakeholders felt OPTIMAL was useful for staff who provide care to residents, with and without ADRD, who need assistance and/or experience behavioral symptoms at mealtime. Further, videotaped observations of dyadic mealtime interactions (N = 265) at three time points supported preliminary efficacy of OPTIMAL: 1) resident mealtime resistive behaviors and functional impairments decreased from T1 to T2 and T3; 2) resident eating independence showed slight decline over time; and 3) resident food intake amount (grams/meal) increased from T1 to T2 and T3.

**Conclusion:**

Findings supported feasibility, acceptability, and usefulness of OPTIMAL in NH staff and residents with dementia. OPTIMAL shows preliminary efficacy in decreasing resident mealtime resistive behaviors and functional impairments, preserving eating independence, and increasing food intake amount in NH residents with moderate‐to‐severe dementia – a vulnerable population that typically experience deteriorations as their cognition declines. Future large‐scale trials are needed to test the efficacy of OPTIMAL in residential care settings.

